# A Novel Homozygous p.L539F Mutation Identified in *PINK1* Gene in a Moroccan Patient with Parkinsonism

**DOI:** 10.1155/2016/3460234

**Published:** 2016-06-20

**Authors:** Rafiqua Ben El Haj, Wafaa Regragui, Rachid Tazi-Ahnini, Asmae Skalli, Naima Bouslam, Ali Benomar, Mohamed Yahyaoui, Ahmed Bouhouche

**Affiliations:** ^1^Research Team on Neurodegenerative Diseases, Medical School and Pharmacy, Mohammed V University, 10100 Rabat, Morocco; ^2^Department of Neurology and Neurogenetics, Specialties Hospital, CHU Ibn Sina, 10100 Rabat, Morocco; ^3^Laboratory of Biotechnology, Medical School and Pharmacy, Mohammed V University, 10100 Rabat, Morocco

## Abstract

Parkinson's disease (PD) is the second most common neurodegenerative disorder after Alzheimer's disease. Ten of fifteen causative genes linked to familial forms of PD have been reported to cause autosomal recessive forms. Among them, mutations in the PTEN-induced kinase 1 (*PINK1*) gene were shown to be responsible for a phenotype characterized by early onset, good response to levodopa, and a benign course. Using chromosomal microarray analysis and Sanger sequencing, we identified a homozygous G/C substitution in a 58-year-old Moroccan man diagnosed with recessive inherited Parkinson's disease. This G-to-C transition occurred at position 1617 leading to an amino acid change L/F at position 539 located in highly conserved motif in the C terminal sequence of* PINK1*. Interestingly, the c.1617G>C substitution is absent in 192 ethnically matched control chromosomes. Our findings have shown that the p.L539F is a novel mutation located in the C terminal sequence of the PINK1 protein that could be pathogenic and responsible for a clinical phenotype resembling idiopathic Parkinson's disease with rapid progression and early cognitive impairment.

## 1. Introduction

Parkinson's disease (PD) is a complex, chronic, and progressive neurodegenerative disease characterized by the classic triad that includes rest tremor, rigidity, and bradykinesia as motor symptoms [1]. The PD prevalence is age-related; it is about 1-2% concerning elderly people over 65 years and can reach 4-5% for subjects over 85 years of age [2]. During the course of disease evolution, secondary symptoms can occur such as motor and nonmotor symptoms leading to a real disability [3]. This symptom array results from a significant dopaminergic neurodegeneration in certain brain areas, especially in the substantia nigra pars compacta [4]. The first PD clinical manifestations occur only when the dopaminergic neurons loss reaches at least 50% [5].

The etiology of PD is likely to be multifactorial involving genetic and environmental factors as well as brain aging process. Nevertheless, several variants in* PARK* loci have been associated with rare Mendelian forms of PD. Among them, there are at least 10 different genes associated with autosomal recessive forms:* PRKN* (PARK2),* PINK1* (PARK6),* DJ-1* (PARK7),* ATP13A2* (PARK9),* PLA2G6* (PARK14),* FBXO7* (PARK15),* DNAJC6* (PARK19), and* SYNJ1* (PARK20) [6] and recently* PODLX* and* VPS13C* genes [7, 8]. Mutations in the* PRKN* gene, including point mutations and exon rearrangements, are the most common and account for up to 50% and 15% of familial autosomal recessive and sporadic early onset PD, respectively.* PRKN* phenotype is indistinguishable from idiopathic PD with good response to levodopa and a benign course [9].* PINK1* gene mutations are the second most frequent in familial early onset PD, compatible with recessive inheritance, and explain 1–9% of cases depending on ethnic origin. The clinical phenotype is characterized by early onset, slow progression, and good levodopa response [10].

Approximately 50 mutations in* PINK1* gene have been considered to be pathogenic in both sporadic and familial PD cases; exon 7 comprises the largest number of mutations found, with Q456X mutation as the most frequent one [11]. Most of these mutations induce a loss of function of the protein, thereby preventing the parkin recruitment and phosphorylation [12]. It is now clear that the combination of the activities of both proteins PINK1 and PRKN is essential for the mitochondrial function regulation and thus for cell survival, by conferring cells neuroprotective properties suggesting critical role of both proteins in the pathogenesis of PD [13].

In the present study, we report a novel homozygous missense mutation in exon 8 of* PINK1* gene in a Moroccan PD patient with a rapid progression and a mild cognitive impairment.

## 2. Patient and Methods

### 2.1. Patient and Controls

The proband, the eldest in a family of five sibs, is a 58-year-old Moroccan man from a consanguineous marriage, referred to the Movement Disorder Unit of the Department of Neurology (Hôpital des Spécialités, Rabat, Morocco) in 2010. Ninety-six control individuals were recruited at the Blood Transfusion Center (Rabat, Morocco). Genomic DNA was extracted from peripheral blood leukocytes using Isolate II Genomic DNA Kit from Bioline. The study was approved by the local ethics committee and an informed written consent was obtained from all participants.

### 2.2. Chromosomal Microarray Analysis

The patient DNA sample was subjected to high resolution CMA using an Affymetrix platform and CytoScan HD microarrays according to the manufacturer's protocol. Briefly, 250 ng of DNA samples was digested with* Nsp1*, amplified with TITANIUM Taq DNA polymerase (Clontech, Mountain View, CA), fragmented with Affymetrix fragmentation reagent, and labeled with biotin end-labeled nucleotides. The DNA was hybridized to the microarray for 16 hours, washed and stained on GeneChip Fluidics Station 450, and scanned on the GeneChip Scanner 3000 7G (Affymetrix). Data analysis was performed using Chromosome Analysis Suite software version 1.2.2 (Affymetrix). These chips include 750,000 SNP and 2.6 million CNV markers that enable high resolution (25–50 kb resolution) detection of CNV, regions of homozygosity (ROH), uniparental disomy, and low-level mosaicism.

### 2.3. Candidate Gene Sequencing

Since the* LRRK2* p.G2019S mutation is very common among PD patients from North Africa and consanguineous patients homozygous for the mutation have been reported [14], exon 41 of* LRRK2* gene was first sequenced in our patient. All the coding exons and intron-exon boundaries of* PINK1* gene were then PCR amplified and the PCR products were sequenced using Big Dye Terminator Cycle Ready Reaction 3.1 Kits and an ABI 3130xl automated sequencer for the patient and the 96 controls. The collected sequence chromatograms were analyzed using SeqScape2.1 software (Applied Biosystems, Foster City, CA).

### 2.4. Bioinformatics Analysis

To predict the pathogenesis of* PINK1* c.1617G>C mutation, the wild and mutant* PINK1* cDNA sequences were both analyzed using MutationTaster software (http://www.mutationtaster.org/). This program calculates probabilities for the alteration to be either a disease mutation or a harmless polymorphism. Similarly, PINK1 amino acid sequences containing L or F at position 539 were analyzed using PolyPhen-2 (http://genetics.bwh.harvard.edu/pph2/) and SIFT software (http://sift.jcvi.org/). Based on straightforward physical and comparative considerations, PolyPhen-2 predicts the possible impact of an amino acid substitution on the structure and function of human protein while SIFT is able to predict whether an amino acid substitution affects protein function.

## 3. Results

### 3.1. Clinical Report

**Figure 1:**
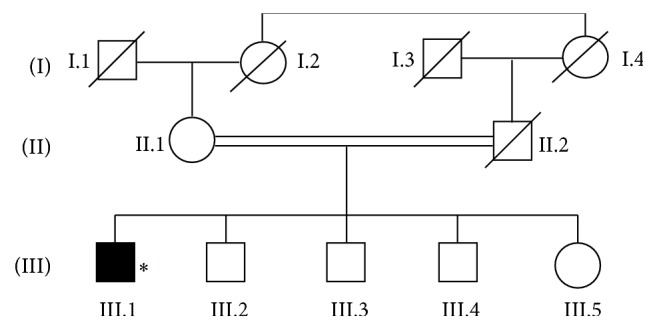
Pedigree of family RBT-BOU-PAR. DNA sample was available from patient with asterisk.

Patient III.1, a 58-year-old man of Moroccan origin, is the eldest in a family of five from a consanguineous marriage of the first degree and without family history of Parkinsonism (Figure 1). His disease began at the age of 54 as a right akinetic-rigid syndrome with slight tremor in the hand. He also complained about neck pain and sleep disturbance (rapid eye movement behavior disorder). He was treated with the dopamine agonist piribedil at 150 mg daily, with good evolution. Levodopa and amantadine were introduced few months later. Two years after disease onset, he complained about morning akinesia and wearing off that resolved with increasing doses of dopaminergic therapy up to 1000 mg of levodopa equivalent dose and 200 mg of amantadine per day. At the age of 58, he had an aggravation of motor fluctuations (delay on and 1 hour of efficacy) and the occurrence of dyskinesia without axial symptoms. The patient was evaluated for deep brain stimulation. MRI and psychiatric evaluation were normal and DOPA test was positive but neuropsychological evaluation revealed a Mattis Dementia Rating Scales score of 128/144. Therefore, a retest was scheduled for 6 months later and therapy adjustment was proposed.

### 3.2. High Resolution CMA

**Figure 2:**
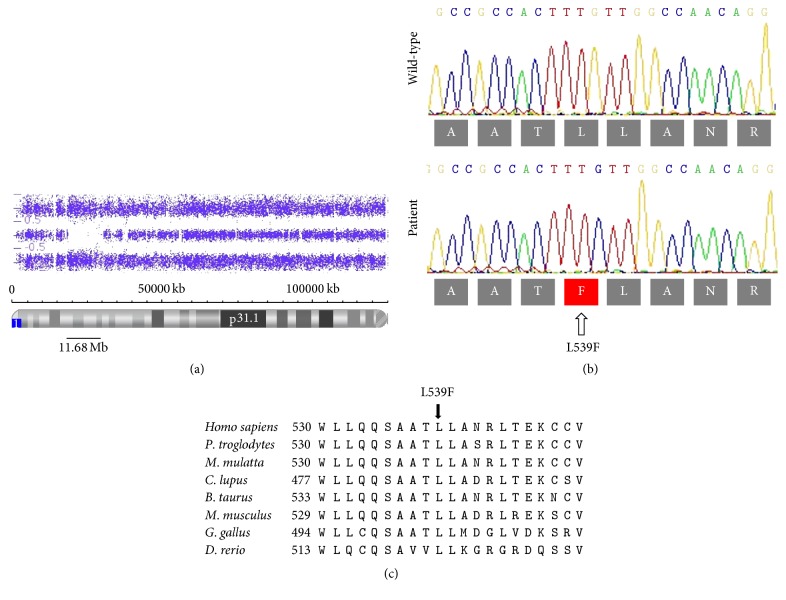
Chromosomal microarray analysis, Sanger sequencing, and sequence alignment for* PINK1 *gene. (a) Allele difference plot of patient III.1 showing a ROH of 11.68 Mb at* PINK1 *gene locus. (b) Sanger sequencing of* PINK1 *exon 8 showing the p.L539F mutation at homozygous state and the wild type sequence in a normal individual. (c) Multiple sequences alignment for human* PINK1 *gene. Conservation of amino acid L at position 539 among various species is indicated by arrow.

Analysis of allele difference plot of the entire genome given by high resolution CMA showed a normal karyotype with no pathogenic CNV but revealed the presence of many ROH through the 23 autosomes. At the 10 known loci for autosomal recessive PD, only one ROH of 11.68 Mb was identified at chromosome 1p from bands 1p36.13 to p35.3 (Figure 2(a)) containing 244 genes including* PINK1* gene.

### 3.3. Candidate Gene Sequencing

Sequencing analysis of exon 41 of* LRRK2* gene did not find the p.G2019S mutation, whereas sequencing of the* PINK1* coding exons and intron-exon boundaries revealed a novel homozygous G-to-C transition (Figure 2(b)) at nucleotide 1617 in* PINK1* exon 8 (c.1617G>C) resulting in a missense mutation of leucine to phenylalanine at amino acid residue 539 (p.L539F). DNA samples of the other family members were unfortunately not available for segregation analysis. However, the p.L539F mutation was absent from 192 ethnically matched control chromosomes. The substituted amino acid, located in the C terminal sequence region outside of the functional domains of the protein, is highly conserved among diverse group of species which indicates the importance of this amino acid. In addition, this mutation is located in a highly conserved amino acid motif from W530 to A541 (Figure 2(c)).

### 3.4. Bioinformatics Analysis

MutationTaster software analysis revealed that* PINK1* c.1617G>C mutation could be a disease causing mutation with a score of 0.977. Analysis with PolyPhen-2 software of p.L539F mutation supports the probably damaging role of this amino acid change with a high score of 0.999 (sensitivity: 0.14; specificity: 0.99). Also, SIFT program gave a probability of 0.02 which predicted a deleterious effect.

## 4. Discussion

In our Moroccan consanguineous PD patient, the whole genome analysis by CMA did not reveal any pathogenic CNV, including exon rearrangements* PRKN* gene, the most common among autosomal recessive forms of PD [9]. However, sequencing of the only ROH-based candidate gene showed a novel homozygous mutation p.L539F in exon 8 of* PINK1* gene. Several evidences were in favor of the pathogenic effect of this novel p.L539F mutation: (1) the patient DNA shows ROH at only* PINK1* locus among the 10 known recessive PD loci; (2) it is absent in 192 ethnically matched chromosomes; (3) it is not reported in publicly available variation databases accessed in February 2016 including dbSNP, ensembl, 1000 Genomes Project, Exome Aggregation Consortium (ExAC), and Exome Sequencing Project (ESP) databases; (4) the mutation affects a highly conserved amino acid motif; and (5) this mutation was predicted to be probably damaging by PolyPhen-2, damaging by SIFT, and disease causing by MutationTaster software. Previously, we have excluded the G2019S mutation of the LRRK2 gene, the most common PD mutation in the Maghreb [14]. Unfortunately, the GBA gene, a worldwide common risk factor for PD particularly in the Ashkenazi Jewish population [15], was not tested in our patient.

PINK1 is a protein of 581 amino acids, with an N terminal mitochondrial targeting sequence, a putative transmembrane domain, a serine/threonine kinase domain, and a C terminal noncatalytic region. The role of the C terminal of the protein is not well known, but it has been postulated that this protein tail could regulate PINK1 kinase activity [16–18] and could play a role in PINK1 targeting and submitochondrial localization [19]. Several* PINK1* mutations have been reported and most of them are located in the protein kinase domain [20]. They are responsible for a clinical phenotype characterized by early onset, generally in the third-fourth decade (range 18–52 years), good response to levodopa, and slow progression but rapid onset of motor fluctuation and dyskinesia. The first symptom is most often tremor or foot dystonia, and additional signs such as sleep disturbance and psychiatric and cognitive impairments could appear during disease evolution [21–23]. In the C terminal tail of* PINK1* gene, only three mutations at homozygous state were reported to be pathogenic. The p.519fsX522 mutation was reported in a patient with an age of onset of 40 years and no particular characteristics in addition to the classical clinical triad of PD [22]. Another frameshift truncating mutation p.D525fsX562 has been found in two patients resembling closely parkin-related disease, with early onset (28 years), good levodopa response, and slow progression [24, 25]. Finally, a small insertion p.534_535insQ, located in the highly conserved amino acid motif (from W530 to A541) 4 nucleotides before our novel mutation, is responsible for early onset PD (32 years) with foot dystonia at onset and a good response to levodopa and without cognitive and psychiatric symptoms after a disease duration of 25 years [26].

The clinical presentation in our patient with this novel homozygous p.L539F mutation was typical with the classical clinical triad of Parkinson's disease but was slightly different from that due to the already reported* PINK1* mutations. Indeed, the disease started relatively later at age 54, the cognitive impairment was early, and the disease progression was rapid since stimulation was proposed for the patient after only 4 years of disease duration.

## 5. Conclusion

We report in this study a novel homozygous missense mutation p.L539F in* PINK1* gene that could be pathogenic in a Moroccan consanguineous patient and extended the phenotypic spectrum of* PINK1*-associated Parkinsonism. Further functional studies should be considered to confirm the pathogenicity of this novel mutation.
